# Copper Metallopolymer Catalyst for the Electrocatalytic Hydrogen Evolution Reaction (HER)

**DOI:** 10.3390/polym11010110

**Published:** 2019-01-10

**Authors:** Sait Elmas, Thomas J. Macdonald, William Skinner, Mats Andersson, Thomas Nann

**Affiliations:** 1 Institute for NanoScale Science & Technology, Flinders University, Bedford Park, SA 5042, Australia; 2 Department of Chemistry, University College London, London WC1H 0AJ, UK; tom.macdonald@ucl.ac.uk; 3 Future Industries Institute, University of South Australia Mawson Lakes Campus, Mawson Lakes, SA 595, Australia; william.skinner@unisa.edu.au; 4 School of Mathematical and Physical Sciences, University of Newcastle, Callaghan, NSW 2308, Australia; thomas.nann@newcastle.edu.au

**Keywords:** copper, metallopolymer, photocurrent, electrocatalysts, hydrogen evolution reaction, p- and n-type photoresponse

## Abstract

Conjugated polymers with stabilizing coordination units for single-site catalytic centers are excellent candidates to minimize the use of expensive noble metal electrode materials. In this study, conjugated metallopolymer, P_OS_[Cu], was synthesized and fully characterized by means of spectroscopical, electrochemical, and photophysical methods. The copper metallopolymer was found to be highly active for the electrocatalytic hydrogen generation (HER) in an aqueous solution at pH 7.4 and overpotentials at 300 mV vs. reversible hydrogen electrode (RHE). Compared to the platinum electrode, the obtained overpotential is only 100 mV higher. The photoelectrochemical tests revealed that the complexation of the conjugated polymer P_OS_ turned its intrinsically electron-accepting (p-type) properties into an electron-donor (n-type) with photocurrent responses ten times higher than the organic photoelectrode.

## 1. Introduction

Conducting and/or conjugated polymers (CPs) are promising materials for organic photovoltaics [1,2,3,4,5]. Beyond their potential use in photovoltaics, CPs are already successfully implemented in many other commercialized electronic devices [6]. Due to their conductive nature, intrinsic semi-conducting properties and the precious advantages of “cheap plastics”, CPs are considered as one of the emerging material classes with much potential, which is not fully explored yet. Especially in the field of metallopolymer or metallosupramolecular polymers, where conducting polymers are complexed with single-site metal centers, there is a lack of research to tap into applications, where the use of metals is indispensable [7]. Organic–inorganic hybrid materials, with new optoelectronic properties, pave the way for potential applications, such as electro and photo catalysts as well as photo electrode materials with improved electronic and optical behaviors [8]. Single-site catalysts in metallopolymers as an alternative to nanoparticulate noble metals can be exposed for photo/-electrocatalysis to make every atom count [9]. Among the conductive/conjugated polymers, the poly(heteroarylene) methines (PHAMs) are well suited to host a series of transition metals into the conjugated polymer backbone. Further to this, their ease of synthesis and structural modification makes them attractive to synthesize metallopolymers for applications of choice. Even though a series of PHAMs polymers are already reported in the literature, their potential applications were mostly limited to photovoltaics, optical devices, and close research fields.

Since the preliminary works of Jenekhe et al. [10] on the polymer class of poly(heteroarylene) methines (PHAMs) in 1986, there has been significant progress on the synthesis and modification of so-called “low-bandgap polymers”. Their peculiar low band-gap [11] makes them highly interesting as materials for organic photovoltaics (OPV) [3] and non-linear applications in photonics [12,13] because they absorb light in the visible range of the spectral light. Their applications in ordered ultrathin films with well-defined architecture using the Langmuir–Blodgett (LB) technique [14] or as sensing material for DNA [15] were also reported. Since PHAMs are known to show tuneable optoelectronic properties, so far most research activities have focused on the substitution effects on pendant heteroaryl units, the aromatic backbone [16,17], and the methylene bridges [18] where immediate influences on the band-gap positions and the band-gap sizes are achieved. Further modifications are known wherein the ratio between aromatic and quinonoidal systems influences the conductivity of the polymeric materials [19,20]. As a consequence of further tailoring of the optoelectronic properties, building blocks with specific properties were incorporated into the poly(heteroarylene) methine backbone, where multistep organic synthesis is involved [21]. Other than that, there is nothing much reported on PHAMs polymers and their metallopolymers at all. 

**Figure 1:**
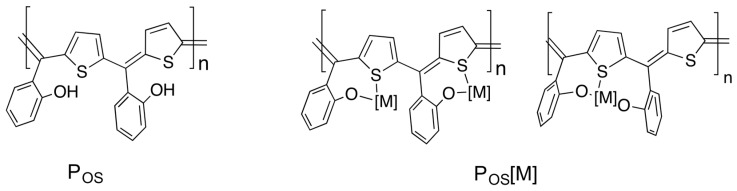
Chemical structures of the poly(heteroarylene) methine polymer P_OS_ and its metal complex P_OS_[M], where the indices OS represent bi- or tridentate coordinating modes and [M] is a metal center with any co-ligands.

We have synthesized a new polymer based on the PHAMs structure and complexed the polymer with copper. Herein, in opposition to the polymer structures known in the literature, the phenoxy functional groups were introduced into the ortho position of the pendant aromatic moieties. Besides the changes in the electronic structure of the polymer itself, the position of the phenoxy group made it possible to introduce transition metals into the repetitive chelating units (Figure 1). The combination of the hard (oxygen) and soft (sulfur) donors that cover the units allows a wide range of transition metals for complexation. Using copper (Cu), the metallopolymer revealed activities for the electrocatalytic hydrogen evolution reaction (HER) at overpotentials comparable to a platinum electrode in a phosphate buffered buffer saline solution (PBS) and at neutral pH 7.4. However, in opposition to the previously reported Cu metallopolymer (Cu MP) with a redox-active hydroquinone/benzoquinone pendant unit [22], the current Cu MP did not show any significant activity towards the oxygen reduction reaction (ORR). To the best of our knowledge, this is the first Cu PHAM electrode material showing activities toward HER at overpotentials close to a Pt electrode and at neutral pH. In addition to the HER activities, the organic/inorganic hybrid material showed an increase of photocurrent and a switch from a p-type (organic) to n-type (hybrid) photo-response under 1.5 air mass (1.5 AM) artificial sunlight, which has not been reported so far. 

## 2. Materials and Methods 

1,4-dioxane (Emsure, Darmstadt, Germany 99.5%), thiophene (Aldrich, St. Louis, MO, USA, ≥99%), salicylaldehyde (Aldrich, 98%), sulfuric acid (Scharlau, Barcelona, Spain, 95–97%), and copper(II) acetate monohydrate (Aldrich) were obtained without further purification. All reactions were performed under ambient conditions at elevated temperatures. 

UV/Vis spectra of the polymer and the copper-metallopolymer were recorded on a Varian UV/Vis spectrometer in acetonitrile using a quartz cuvette with an optical path length of 1.0 cm. 

X-ray photoelectron spectroscopy (XPS) was performed with a Kratos Ultra DLD spectrometer, using monochromatic Al kα radiation (hν = 1486.7 eV). The system is equipped with a magnetically confined charge compensation system (low energy electrons are confined and transported to the sample surface by a magnetic field). Spectra were recorded using an acceleration voltage of 15 keV at a power of 225 W. Survey spectra were collected with a pass energy of 160 eV and an analysis area of 300 μm × 700 μm. Data analysis was performed with CasaXPS software (Casa Software Ltd.) and selected graphs were plotted with the Qti Plot software.

Cyclic voltammograms were recorded in a three-electrode configured electrochemical cell on an AUTOLAB potentiostat. A platinum rod was used as a counter electrode (CE) and the deposited films on gold substrates acted as working electrodes (WE). All recorded currents are referred to as Ag|AgCl (3 M). For the fabrication of the working electrodes gold substrates (100 nm Au with 40 nm Ti sublayer on microscope slides, obtained from RDLI Inc.) were coated with the polymer P_OS_ and P_OS_[Cu] respectively. Hydrogen evolution reactions (HER) were recorded on an AUTOLAB potentiostat using potentiostatic cyclic voltammetry methods. Here, the materials P_OS_ and P_OS_[Cu] were drop-casted on a gold substrate with 1 × 1 cm^2^ surface area acted as the working electrode (WE), each. A platinum rod was used as a counter electrode (CE) and Ag|AgCl (3 M KCl) acted as reference electrode (RE). The cyclic voltammograms (CV) were recorded at 100 mV/s in the potential range of 0.2 to −1 volts vs. Ag|AgCl (3 M KCl). For better comparisons, the recorded working potentials vs. Ag|AgCl (3 M KCl) were converted into the reversible hydrogen electrode (RHE) according to the equation E_(RHE)_ = E_(Ag|AgCl)_ + 0.059·pH + 0.210 V, where pH of 0.1 M PSS was 7.4.

Hydrogen was measured by gas chromatography using a Hewlett Packard 5890 series II GC with a thermal conductivity detector (TCD) and employing a molecular sieve 5 A (80–100 mesh) 2 m column run at 60 °C with argon as the carrier gas.

### 2.1. Synthesis of P_OS_ and P_OS_[Cu]

*Poly(thiophene-2,5-diyl)(o-hydroxybenzylidene)*, *P_OS_*: Thiophene (2 mL, 2.1 g, 25 mmol), o-hydroxybenzaldehyde (2.96 mL, 3.39 g, 27.75 mmol) and 0.5 mL sulphuric acid (97%) were dissolved 10 mL 1,4-dioxane and refluxed for 20 h at 80 °C. The formed black polymer was precipitated by adding 20 mL methanol/water mixture (1/1) and washed twice with cold methanol/water (1/1). The black solid was then re-dissolved in THF and transferred into a round-bottom flask. After removal of all solvents and volatiles on the rotary evaporator, the polymer was obtained as black, crystalline powder (3.5 g).

*Polymer complex with Cu(II) acetate, P_OS_[Cu]*: The polymer P_OS_ (1 g, 5.33 mmol referred to the MW of one repetition unit) and copper(II) acetate monohydrate (1 g, 5.01 mmol) were dispersed in 20 mL MeOH and refluxed for 24 h at 60 °C. After removal of all volatiles and solvents on the rotary evaporator, the brown fine powder was washed 3 times with THF and dried in vacuum. The copper metallopolymer was obtained almost quantitatively as a brown fine powder, which is sparingly to non-soluble in most common organic solvents. 

### 2.2. Electrode Preparation

*Polymer P_OS_[Cu] spin-coat deposition*: P_OS_ films were prepared using a Laurell WS-650S-6NPP/LITE spin coater (North Wales, PA, USA). The spin-coating solution consisted of 5 mL terpineol, 200 mg of the metallopolymer, and 5 drops acetylacetone. The P_OS_[Cu] was spin-coated onto a gold surface at 500 RPM for 10 s, followed by ramping to 2000 RPM for 30 s. These parameters resulted in a uniform coating of P_OS_[Cu] across the gold surface. Terpineol was then evaporated using an O_2_ furnace at 250 °C for 30 min. The heating rate was set to 5 °C/min. The P_OS_ film thickness was confirmed to be ~100 μm using a profilometer (Bruker, Billerica, MA, USA, Dektak XT). The spin-coated P_OS_[Cu] was mounted into the photoelectrochemical (PEC) for the chronoamperometric tests under 1.5 AM artificial sunlight.

*Drop-casting of P_OS_ and P_OS_[Cu] for electrocatalytic tests (HER)*: P_OS_ and P_OS_[Cu] (10 mg) were dispersed in 20 mL iso-propanol (iProH) and sonicated for 30 min in an ultrasonic bath (Fisher Scientific FB15047), each. The dispersion was then slowly dropped onto the surface of gold substrates and the substrates were heated from the rear side with a heating gun until a clear black film was formed on the surface. The substrates were held with tweezers at a distance of 15–20 cm from the heating gun to avoid overheating of the films. The temperature of the heating gun was set to 150 °C. To obtain reasonable films the dropping sequence was adjusted to the complete evaporation of previous droplets. Occasionally the substrates were slightly tilted up and down to focus the evaporating droplets to vacant surface areas. The resulting polymer and metallopolymer electrodes were then air-dried and the active surface area was limited to 1.5 × 1.5 cm^2^ covered with film by wiping off the excess of film with KimTech Wipes moistened with iPrOH. The prepared electrodes were then mounted immediately into the electro-chemical cell for electrocatalytic HER tests. 

## 3. Results and Discussion

### 3.1. Synthesis and Characterization of P_OS_ and P_OS_[Cu]

For the synthesis of the polymer P_OS_, we followed the protocol reported by Jenekhe [19,23]. Typically, thiophene, the aromatic aldehyde, and catalytic amounts of sulfuric acid were added in a one-pot-reaction and the mixture was refluxed overnight. After removing the solvents and the volatiles the polymer was obtained as black chunky solid and analyzed by UV/Vis, cyclic voltammetry (CV), and XPS. The polymer was slightly soluble in common organic solvents allowing purification by washing out unreacted starting materials and oligomer residuals. The best solubility was observed in dimethyl sulfoxide (DMSO) or acetonitrile (AN). The Cu metallopolymer was obtained as a dark brown, fine powder. As a consequence of the higher molecular weight, the metallopolymer became comparably poorly soluble in organic solvents indicating successful complexation.

**Figure 2:**
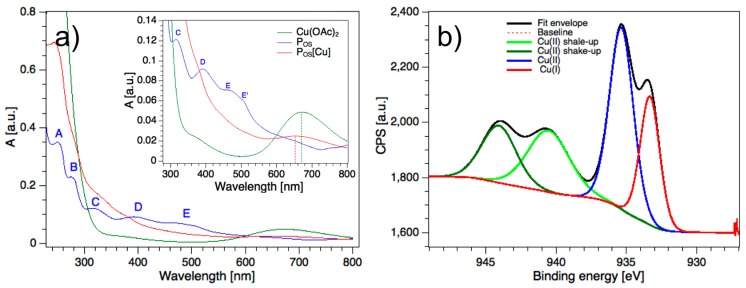
(**a**) UV/Vis absorption spectra of the polymer P_OS_ and its coordination compound P_OS_[Cu] (inset) in acetonitrile; (**b**) binding energies of the Cu 2p core levels analyzed by XPS.

The UV/Vis absorption spectra of the polymer P_OS_ and its complex compound with Cu(II) were recorded in AN. For comparisons, the Cu(II) acetate precursor was recorded in the same solvent. As depicted in Figure 2a the polymer P_OS_ shows five distinct absorption peaks (A 250 nm, B 276/286 nm, C 317 nm, D 393 nm, E 470/504, where two of them are in the range of the visible light. Among them, the absorption bands B and E appear with a shoulder, each. 

After complexation with Cu(OAc)_2_ the transitions of the resulting metallopolymer, P_OS_[Cu], showed slight hypsochromic shifts in the UV range indicating ligand (polymer)-to-metal charge transfer effects (LMCT). It appears that the complexation caused significant quenching effects on the transitions at 393 nm (D) and 470/504 nm (E) in the visible region (inset in Figure 2a). The broad and weak absorption band in the range of 600–700 nm is assigned to the d–d transitions of copper complexes [24], which is—compared to the Cu(OAc)_2_—slightly shifted to lower wavelengths (Figure 2a, inset).

The chemical states of the polymer P_OS_ and its metallopolymer P_OS_[Cu] were analyzed by X-ray photoelectron spectroscopy (XPS). Figure 2b shows the binding energies (BE) of the 2p electrons of the copper centers in the polymer P_OS_[Cu]. The binding energies of P_OS_[Cu] showed that the metallopolymer exhibited 22% of Cu(I) species with a remarkably high fraction of Cu(II) shake-up satellites (40%). The shake-up peaks in transition metals complexes are discussed as indicative charge-transfer effects between metal and ligands [25,26]. A similar polymer based on the same PHAM structure, the poly(isonaphthalene) methine reported by Sariciftci et al. [27] spontaneously reacts with molecular oxygen leading to post-oxidized polymers. Here, the post-oxidation of the polymer was possibly triggered by the redox chemistry of the copper, which remained as a reduced species in the polymer. Copper is known to be partly reduced by x-ray radiation under ambient conditions during the sample analysis [28] that can also be assisted by ligand effects. Nonetheless, the high content of Cu(I) follows the trend of the previously reported Cu metallopolymer with a redox-active moiety [22].

**Figure 3:**
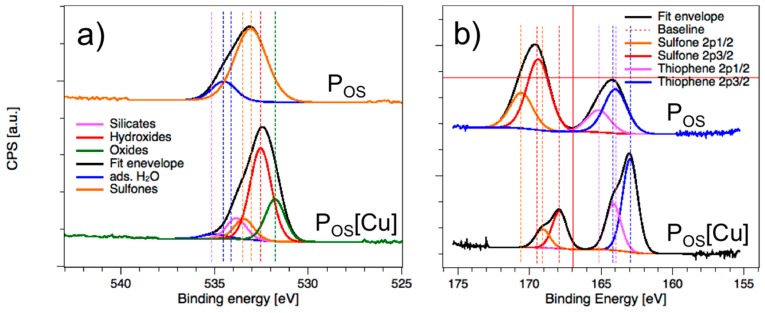
(**a**) O(1s) and (**b**) S(2p) core-level spectra of the analyzed samples P_OS_ (top) and P_OS_[Cu] (bottom) by X-ray photoelectron spectroscopy.

The O(1) core level spectrum in the P_OS_ sample (Figure 3a) exhibited a broad sulfone (R_1_R_2_SO_2_) fitting centered at 533.0 eV and strongly overlapping the phenol and other O-functional groups, which then became much less pronounced in the P_OS_[Cu] material (533.4 eV). In the latter sample, phenol/phenoxy O(1s) appeared as dominating functional groups in the fit envelope which were centered at 532.5 eV. The oxides centered at 531.7 eV and appearing as the second dominating fitting originated from oxygen attached to metals indicating complex formation (P_OS_[M], Figure 1). 

The S(2p) core-level spectra of the polymer P_OS_ and its metal hybrid P_OS_[Cu] exhibited two distinct binding energies with different ratios in intensity indicating mixtures of thiophene and sulfone functional groups (Figure 3b), each [22,29,30]. In both materials, the fitted binding energies appeared with the typical S(2p) doublet with 2/1 ratio and 1.2 eV splitting [30]. However, the most interesting feature of the S(2p) core level spectra is the change in the intensities from sulfone functional group being the major species in P_OS_ to the thiophene in P_OS_[Cu] and the significant shift to lower binding energies for both groups. The reduction of the sulfone content in S(2p) after complexation is consistent with the trend observed in the O(1s) core level data. After complexation with metal, the S(2p) doublets (S2p3/2 and S2p1/2) appeared more distinctive in the envelope showing much narrower width of the fittings in total (P_OS_[Cu], Figure 3b).

In total, all atoms in P_OS_[Cu] involved in the coordination of the copper centers were at about 1–2 eV lower in their binding energies compared to those obtained from the pure polymer indicating electronic impacts from the copper centers. The atomic ratio of 3.45/2.05 between sulfur and copper in P_OS_[Cu] survey spectrum (Figure S1, supporting information) is more indicative for a tridentate coordination mode (Figure 1) around the Cu centers. The tridentate mode becomes more clear if we consider 78% of the available copper in the oxidation +2, which then results in a S/Cu(II) ratio of 2.16/1.0 per repetition unit.

### 3.2. Photophysical Properties

The photocurrent measurements of the pure polymer P_OS_ and its metallopolymer P_OS_[Cu] were undertaken in a photoelectrochemical cell (PEC) using a platinum wire as a counter electrode (CE) and Ag|AgCl (3 M KCl) as a reference electrode (RE). P_OS_ and P_OS_[Cu] deposited on a gold substrate acted as working electrodes (WE). For the deposition of the samples, we conducted two methods: The spin-coating technique and deposition via doctor blading method. The latter being a technique we already conducted for the deposition of inorganic metal oxides [31] in our previous work on photoelectrode fabrication. We found spin-coating to be the better option for this work due to the viscosity of the samples. Due to the varying solubility, different film thicknesses were obtained. The film thickness of P_OS_[Cu] was found to be 100 nm (±10 nm) by ellipsometry methods, where the sample of the pure polymer could not be examined due to the higher thickness. Their photocurrent responses were recorded in a 0.1 M phosphate-buffered saline solution (PBS, pH 7.4) under dark conditions and illumination with 1.5 AM artificial solar light at their open circuit potentials.

**Figure 4:**
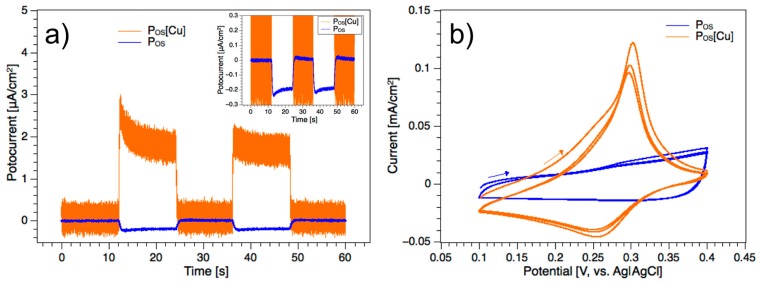
(**a**) Photocurrent response of the polymer and its copper complex in a 0.1 M saline solution; the photocurrents were recorded in the darkness for the first 12 s (s) followed by another 12 s under 1.5 AM artificial sunlight and again for 12 s darkness and 12 s light exposure, respectively; (**b**) cyclic voltammogram of both samples in 0.1 M KCl electrolyte solution in the potential range of 0.1–0.4 V and at a scan rate of 50 mV/s.

Figure 4a shows the chronoamperograms of the polymer P_OS_ and its metallopolymer P_OS_[Cu] for the first 12 s under dark conditions and illumination with 1.5 AM artificial solar light (12 s). The current responses were recorded for 60 s under dark conditions and illumination for 12 s, each. The P_OS_ electrode showed a negative p-type response of 0.26 μA/cm^2^ (inset, Figure 4a) whereas the P_OS_[Cu] electrode showed an opposite n-type (4a) behavior of 2.6 µA/cm^2^ under the same conditions. The complexation of the conjugated polymer with copper turned the behavior of an intrinsically electron-accepting material (p-type) to an electron-donor (n-type). Although the thickness of the Cu(II)-doped film was much lower than the pure polymer film, the positive current density was, remarkably, 10 times higher than the current response of the pure polymer. Both electrodes respond with a sharp peak current (Figure 4a) that decays slowly during the turn on phase (photocurrent transients). The effect of photocurrent transient has been assigned to trapping of charges in the film and/or charge-accumulation at the semi-conductor-liquid junction [32,33,34]. 

The electron donor behavior of P_OS_[Cu] and the results from XPS were corroborated by electrochemical investigations as it revealed copper centers in the oxidation state I. Figure 4b shows the cyclic voltammograms of P_OS_ and P_OS_[Cu] in a 0.1 M KCl solution. The copper complex shows a strong oxidation event at 0.29 volts vs. Ag|AgCl (3 M KCl) which is assigned to the redox couple Cu(I/II), whereas no redox peaks could be observed in the same potential range for the polymer. It is known that metal doping increases the photocurrent response of CPs [35,36]. However, to the best of our knowledge, the change of the photophysical behavior of CPs from p-type to n-type photoresponse in conjunction with metal complexation has not been reported yet. 

### 3.3. Electrocatalytic Hydrogen Evolution Reaction (HER)

**Figure 5:**
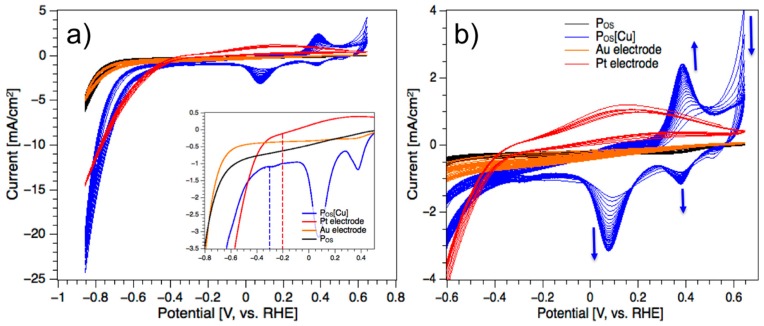
(**a**) Cyclic voltammogram of P_OS_[Cu] in 0.1 M PBS solution at a scan rate of 100 mV/s and 30 number of potentiometric sweeps compared to the polymer P_OS_ (16 sweeps), the Au substrate (18 sweeps) and the Pt electrode (7 sweeps); (**b**) cut-out of the CV from 5a highlighting the reversible redox peaks between 100 and 400 mV vs. RHE.

Earth abundant transition metals and their molecular structures are of great interest because of their potential to replace expensive platinum-group metals in the photo/-electrocatalytic water splitting [37,38,39,40]. The P_OS_ and P_OS_[Cu] electrodes were placed in an electrochemical cell and tested for the electrocatalytic hydrogen generation (HER). Here, P_OS_ and P_OS_[Cu] were drop casted on gold substrates and swept in 0.1 M PBS solution between 0.65 and −0.85 V vs. RHE. Their catalytic activities for HER were compared to a platinum disk electrode and a bare Au substrate. As shown in Figure 5a, the cyclic voltammograms of the gold and polymer electrodes are similar and the obtained peak current densities were at around −5 mA/cm^2^ at −0.85 V, both. During the potentiometric sweeping, slight gas bubbles were observed at the polymer and gold electrodes at −0.85 V vs. RHE. Since the gold electrode [41,42] is known to act as a HER catalyst itself, the generated hydrogen at the polymer electrode is most likely caused by the gold under layer. Nonetheless, the copper metallopolymer electrode P_OS_[Cu] showed a much higher gas evolution rate at the working electrode. The hydrogen reduction was already initiated at a low overpotential of −300 mV vs. RHE (inset, Figure 5a) and exhibited a peak current density of −25 mA/cm^2^ at −850 mV, which is five times higher than what was obtained at the metal free and gold electrodes (Figure 5a). The evolved H_2_ was confirmed by GC analysis (Figure S2, supporting information). After 30 subsequent potentiometric sweeping, no polymer leaching or significant decreases in current density could be observed. Additionally, as shown in Figure 5b, a reversible redox event appeared between 100 and 400 mV vs. RHE, which was not observed at the pure polymer electrode. The reversible redox peak is assigned to the Cu(I/II) species. Notably, the reversible redox event became more intensive and sharp with the increasing number of the potentiostatic sweeps indicating generation of more active redox species within the subsequent number of sweeps. 

For comparison, a platinum disk was used under the same conditions. The hydrogen generation was initiated at −200 mV vs. RHE and the generated current density steadily increased up to 15 mA/cm^2^ at the lower end potential. In the potential window between the onset reduction potential of −200 and −670 mV, it demonstrated higher performance than P_OS_[Cu], but it was then overtaken by the metallopolymer catalyst at increased anodic overpotentials (Figure 5). The comparably lower peak current density at higher overpotentials is most likely caused by residing gas bubbles at the flat electrode surface causing a reduction of active surface area. This effect is overcome in the metallopolymer electrode because of the naturally porous structure of the hybrid catalyst and higher accessibility of the coordination sites for the water molecules.

At neutral pH, an efficient copper molybdenum sulfide electrocatalyst was reported to generate molecular hydrogen (H_2_) at a reduction potential of −160 mV vs. RHE [43], which is 140 mV lower than the overpotential of the current P_OS_[Cu] catalyst. The high activity of the reported Cu_2_MoS_4_ electrocatalyst, which mimics the active sites of the molybdenum CO-dehydrogenase, is associated with two possible intermediate states facilitating spontaneous H_2_ evolution during the one-electron-reduction. However, the onset potential of P_OS_[Cu] for the H_2_ production is comparable to transition metal sulfide [44] and phosphide [45] catalysts, but is lower than the reduction potentials of protons obtained at, i.e., metal-free electrocatalysts [46], transition metals on carbon materials such as NiWS/CF [47] and Ni2P/CNS [48], or molecular catalysts [49,50,51]. To obtain a current density of 10 mA/cm^2^, the P_OS_[Cu] and the blank Pt electrodes require an overpotential of 760 mV vs. RHE (Figure 5a) in neutral media, both. This overpotential is basically comparable to the efficient electrocatalysts Cu_2_MoS_4_ [43] and Ni-S [44] to obtain the same current density under same pH conditions.

## 4. Conclusions

To summarize, the combination of transition metals with conjugated/conducting polymers enables the exploration of hybrid organic/inorganic materials with manifold potential applications, which are yet to be explored. In the electrocatalytic hydrogen evolution reaction (HER), the copper-based metallopolymer reduced protons at a neutral pH and at overpotentials, which are only by 100 mV higher than the blank Pt electrode. To obtain a current density of 10 mA/cm^2^ in the HER, the P_OS_[Cu] catalyst requires overpotentials which are comparable to efficient platinum-free catalysts. The metallopolymer electrode showed stable performance after 30 sweeps without adding sacrificial agents and acidifying the electrolyte solution. The combination of a soft and easily processable conducting polymeric backbone with molecular catalyst enables the designing of new electrode materials made of well-defined single-site catalysts. The photophysical investigations of the P_OS_[Cu] metallopolymer revealed a change of initially p-type photoresponse of the organic polymer to n-type behavior. The obtained current densities at the P_OS_[Cu] photoelectrode were ten times higher than the metal-free one thus enabling new potentials in the design of photocatalysts.

## Supplementary Materials

The following are available online at http://www.mdpi.com/2073-4360/11/1/110/s1, Figure S1: XPS survey spectrum of the polymer sample P_OS_. Figure S2: XPS survey spectrum of the metallopolymer sample P_OS_[Cu]. Figure S3: (a) Gas chromatogram of the reference gas (200 ppm H_2_) and (b) gas chromatogram obtained from the head-space during the HER.
