# Supplementary material for: Copper Metallopolymer Catalyst for the Electrocatalytic Hydrogen Evolution Reaction (HER)
Source: Polymers (Basel). 2019 Jan 10;11(1):110. doi: 10.3390/polym11010110 (PMC6401685; doi:10.3390/polym11010110)

Electronic Supporting Information

Copper Metallopolymer Catalyst for the Electrocatalytic Hydrogen Evolution Reaction (HER)

Sait Elmas* [1], Thomas J. Macdonald [2], William Skinner [3], Mats Andersson* [1], and Thomas Nann[4]

[1] Institute for NanoScale Science & Technology, Flinders University, Bedford Park SA 5042, Australia; sait.elmas@flinders.edu.au, mats.andersson@flinders.edu.au.
[2] Department of Chemistry, University College London, WC1H 0AJ, United Kingdom; tom.macdonald@ucl.ac.uk.
[3] Future Industries Institute, University of South Australia Mawson Lakes Campus, Mawson Lakes SA 595, Australia; william.skinner@unisa.edu.au.
[4] School of Mathematical and Physical Sciences, University of Newcastle, Callaghan NSW 2308, Australia; thomas.nann@newcastle.edu.au.
* Correspondence: sait.elmas@flinders.edu.au; Tel.: +61-8820-12684

Table of content

Figure S1: XPS survey spectrum of the polymer sample P_{OS}……………………………………………………S2

Figure S2: XPS survey spectrum of the metallopolymer sample P_{OS}[Cu]……………………………………………S2

Figure S3: (a) Gas chromatogram of the reference gas (200 ppm H_2) and b) gas chromatogram obtained from the head-space during the HER……………………………………………………………………………S3

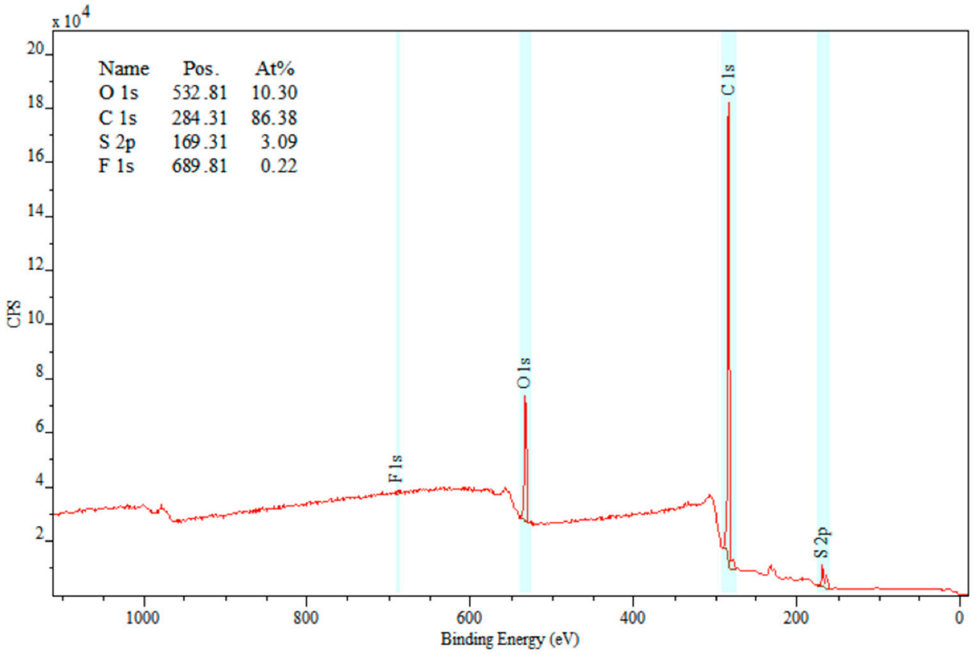

Figure S 1: XPS survey spectrum of the polymer sample $P_{OS.}$

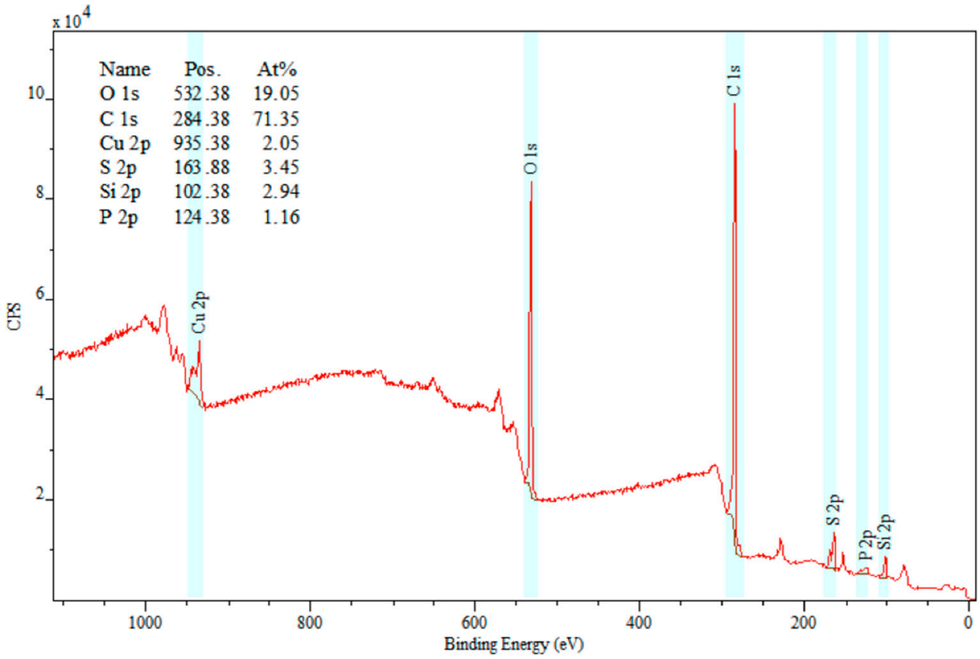

Figure S 2: XPS survey spectrum of the metallopolymer sample P_{OS}[Cu].

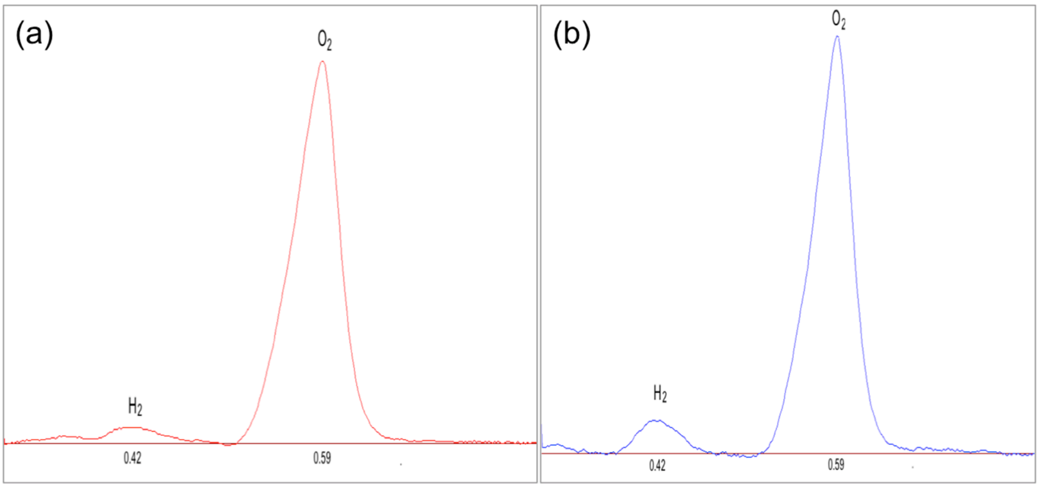

Figure S 3: (**a**) Gas chromatogram of the reference gas (200 ppm H_2) and (**b**) gas chromatogram obtained from the head-space during the HER.

Pictorial:

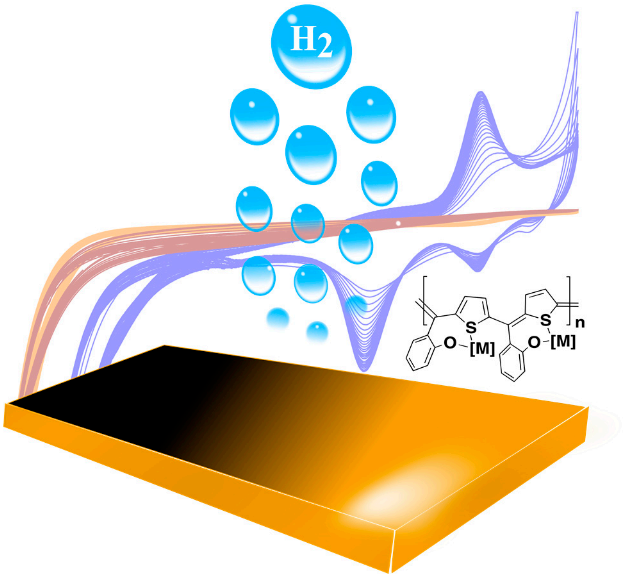

Supplement: Supplementary file 1 [file polymers-11-00110-s001.pdf]
